# Fully automated segmentation and classification of renal tumors on CT scans via machine learning

**DOI:** 10.1186/s12885-025-13582-6

**Published:** 2025-01-29

**Authors:** Jang Hee Han, Byung Woo Kim, Taek Min Kim, Ji Yeon Ko, Seung Jae Choi, Minho Kang, Sang Youn Kim, Jeong Yeon Cho, Ja Hyeon Ku, Cheol Kwak, Young-Gon Kim, Chang Wook Jeong

**Affiliations:** 1 Department of Urology, Seoul National University Hospital, 101 Daehak-ro, Jongno-gu, Seoul, 03080 South Korea; 2 Department of Urology, Seoul National University College of Medicine, 101 Daehak-ro, Jongno-gu, Seoul, 03080 South Korea; 3 Department of Transdisciplinary Medicine, Seoul National University Hospital, 101 Daehak-ro, Jongno-gu, Seoul, 03080 Republic of Korea; 4 Department of Radiology, Seoul National University Hospital, 101 Daehak-ro, Jongno-gu, 03080 Seoul, Republic of Korea; 5 Department of Radiology, Seoul National University College of Medicine, 101 Daehak-ro, Jongno-gu, 03080 Seoul, Republic of Korea; 6 Department of Medicine, Seoul National University College of Medicine, 101 Daehak-ro, Jongno-gu, Seoul, 03080 Republic of Korea

**Keywords:** CT, Renal cell carcinoma, Radiomics, Machine learning, Deep learning, Segmentation, Classification

## Abstract

**Background:**

To develop and test the performance of a fully automated system for classifying renal tumor subtypes via deep machine learning for automated segmentation and classification.

**Materials and methods:**

The model was developed using computed tomography (CT) images of pathologically proven renal tumors collected from a prospective cohort at a medical center between March 2016 and December 2020. A total of 561 renal tumors were included: 233 clear cell renal cell carcinomas (RCCs), 82 papillary RCCs, 74 chromophobe RCCs, and 172 angiomyolipomas. Renal tumor masks manually drawn on contrast-enhanced CT images were used to develop a 3D U-Net-based deep learning model for fully automated tumor segmentation. After segmentation, the entire classification pipeline, including feature extraction and subtype classification, was conducted without any manual intervention. Both conventional radiological features (Hounsfield units, HUs) and radiomic features extracted from areas predicted by the deep learning models were used to develop an algorithm for classifying renal tumor subtypes via a random forest classifier. The performance of the segmentation model was evaluated using the Dice similarity coefficient, while the classification model was assessed based on accuracy, sensitivity, and specificity.

**Results:**

For tumors larger than 4 cm, the Dice similarity coefficient (DSC) for automated segmentation was 0.83, while for tumors smaller than 4 cm, the DSC was 0.65. The classification accuracy (ACC) for distinguishing RCC subtypes was 0.77 for tumors larger than 4 cm and 0.68 for tumors smaller than 4 cm. Additionally, the accuracy for benign versus malignant classification was 0.85.

**Conclusions:**

Our automatic segmentation and classifier model showed promising results for renal tumor segmentation and classification.

**Supplementary Information:**

The online version contains supplementary material available at 10.1186/s12885-025-13582-6.

## Background

Up to 70% of renal tumors are detected incidentally by imaging [1], which has led to uncertainty among clinicians as to which tumors should be promptly resected. Accordingly, accurate classification and subtyping of renal masses are becoming increasingly important. In this context, two major issues need to be addressed to improve patient outcomes, namely, differentiating between benign and malignant tumors, particularly small renal masses (SRMs), and distinguishing the renal cell carcinoma (RCC) subtype.

When considering the differentiation of benign and malignant SRMs, it is important to note that up to 20% of lesions are pathologically benign [2]. The second issue involves distinguishing RCC subtypes, namely, clear cell RCC, with an incidence of approximately 70%, from subtypes that have a lower incidence rate, namely, papillary (10–20%) and chromophobe (5–10%) RCCs [3]. This differentiation is important, as these different subtypes have different prognoses and molecular targeted therapies. Renal biopsy can be helpful in achieving this differentiation; however, biopsy can cause various unpredictable risks, such as metastasis of cancer cells, tumor hemorrhage, and sampling errors [3].

Previous studies utilizing manual segmentation of computed tomography (CT) imaging with conventional radiomic features have demonstrated the potential of radiomics for renal tumor classification [4–6]. However, these approaches may lead to data inconsistencies due to their manual nature, and clinically relevant imaging findings might not have been fully captured. To address these challenges, our study aimed to investigate the feasibility of a fully automated 3D U-Net-based deep learning model for segmenting renal and tumor regions. This model was developed using data from a prospectively collected cohort, which was carefully designed and clinically validated to enhance consistency and reliability. Furthermore, we propose that incorporating Hounsfield unit (HU) values alongside radiomic features may improve the accuracy of renal tumor classification.

The primary objective of this study was to explore the potential of developing and validating a fully automated system that could differentiate between benign and malignant tumors, as well as distinguish RCC subtypes. By leveraging a robust dataset and examining the integration of HU values with radiomic features, this approach seeks to improve the reliability of the model and provide a foundation for clinical applicability.

## Materials and methods

**Fig. 1:**
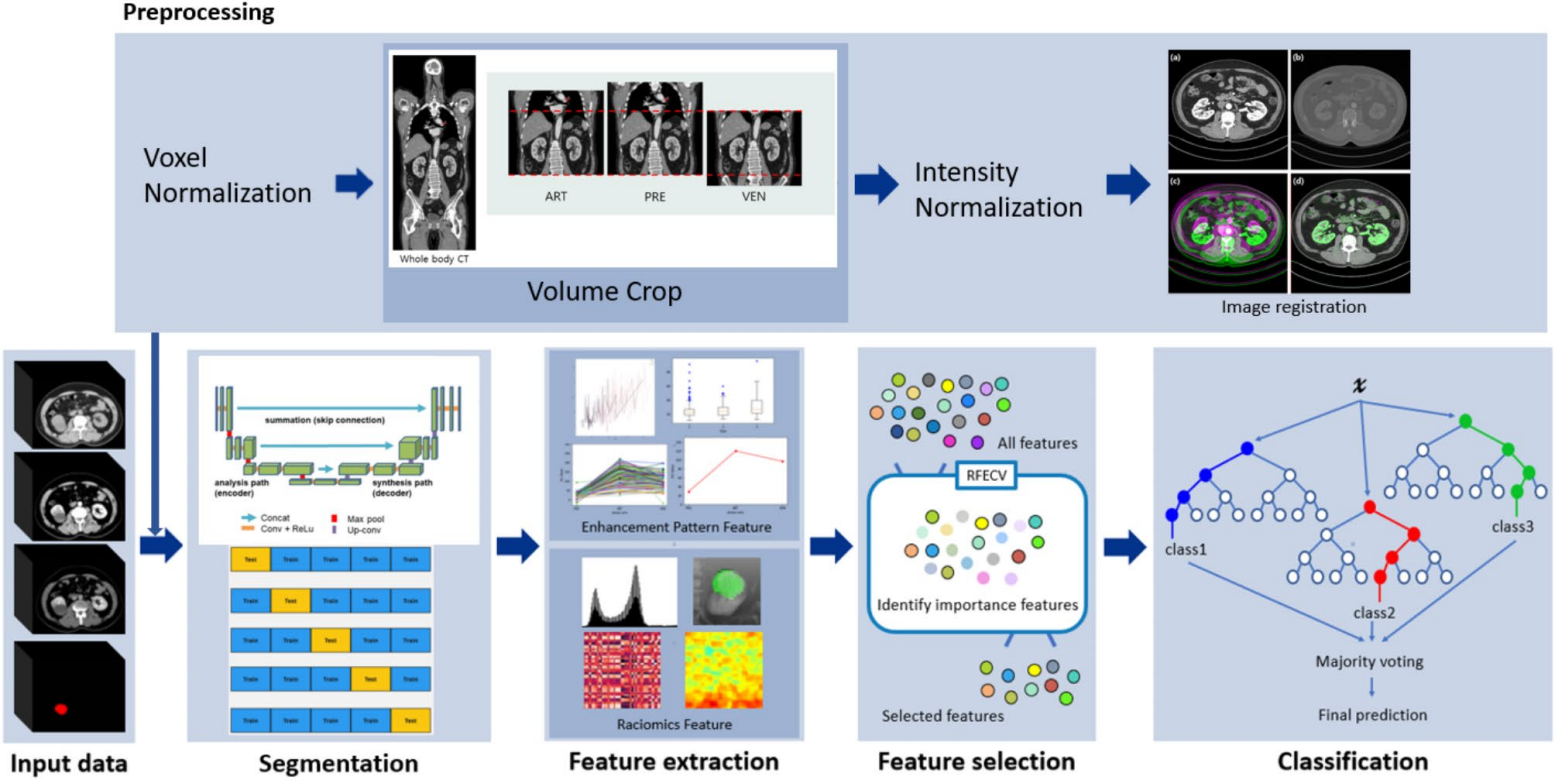
Flow-through of the automated segmentation and classification pipeline

The framework of this study is shown in Fig. 1.

### Study population

We reviewed preoperative CT images and medical records of patients registered in a prospectively collected database, the Seoul National University Prospectively Enrolled Registry for RCC– Nephrectomy (SUPER-RCC-Nx) [7], who underwent partial or radical nephrectomy at a medical center between March 2016 and December 2020. The exclusion criteria were as follows: (1) low-quality CT images; (2) a cystic renal mass; (3) benign pathology, other than angiomyolipoma (AML); and (4) a recurrent tumor or multiple tumors of the ipsilateral kidney. Patient demographics, distributions of kidney tumor subtypes, and tumor sizes used for the training and testing datasets are summarized in Table 1. After selection, CT images of 561 renal masses were eligible for the following two models. For the RCC subtype classification model, CT images of 389 pathologically proven RCCs (233 clear-cell, 74 papillary, and 82 chromophobe RCC types) were used. For the benign/malignant classification model, CT images of 391 small renal masses (SRMs) ≤ 4 cm in size were used.

**Table 1:** Baseline characteristics of patients whose images were used for the training and test datasets

	Total	Training	Test
	No	No	No
**Patient (n)**	561	448	113
**Age (y)** ^**a**^	56 ± 12.7	56 ± 12.4	53 ± 13.4
**Sex**			
Male	293	229	64
Female	268	219	49
**Tumor Diameter (cm)**			
≤ 4.0^b^	391	313	78
Other^c^	170	135	35
**Tumor Histological Type**			
Clear cell	233	186	47
Chromophobe	74	59	15
Papillary	82	66	16
Angiomyolipoma(AML)	172	137	35
**Location**			
Left	288	227	61
Right	263	211	52
Both	10	10	0

^a^ Values are expressed as mean ± standard deviation

^b^ All histology types are included

^c^ Only renal cell carcinoma (RCC) types are included

y, years

### CT examination

All CT imaging was performed with patients in the supine position, with images acquired during a single breath-hold to minimize motion and misregistration artifacts. Images were obtained in slices from the superior aspect of the diaphragm to the inferior margin of the symphysis pubis. Images were obtained via a multidetector CT scanner (64–128 channels) in three distinct phases: precontrast, late arterial (corticomedullary), and delayed (excretory) phases, using 80–120 kVp tube energy, 45 s and 180 s after contrast medium administration. The imaging was performed with the following parameters: collimation of 64 × 0.625 mm, 64 × 0.6 mm, or 128 × 0.6 mm; gantry rotation time of 0.5 s; pitch of 0.891 or 0.65; slice thickness of 2.5–5 mm; reconstruction interval of 2.5–5 mm; and matrix size of 512 × 512. Iodinated contrast medium, with a concentration of 350 mg/mL, was administered into the peripheral vein of the upper extremity via an automatic power injector at a total dosage of 1.5 mL/kg over 30 s.

### Data preprocessing

Two experienced radiologists manually annotated the renal tumors for each imaging phase using the commercially available software MEDIP PRO (MEDICAL IP, Seoul, Republic of Korea), with annotations being cross-validated between them to ensure accuracy and consistency. To ensure data consistency, several preprocessing steps were carried out, including format conversion, intensity normalization, resampling, and registration. First, the annotated CT images and labeled masks were cropped to the same plane and resampled to a voxel size of 1 × 1 × 1 mm (resulting in a resolution of 256 × 256 × 256 pixels) using spline interpolation to preserve regional information. Subsequently, the resampled images were then registered for the precontrast and delayed phases, with the arterial phase used as the fixed image for each case, as previously described, using Elastix open-source software [8]. Additionally, intensity normalization was performed to standardize the data, with the window level and width set to 40 and 400 HU, respectively, to remove irrelevant information and background noise.

### Data splitting

The number of tumor subtypes was imbalanced; therefore, we employed a 5 fold data splitting strategy. Specifically, the entire dataset was strategically partitioned into five distinct subsets to ensure a representative distribution of each tumor subtype across the sets. By training the classification model on each of the five datasets, we obtained five distinct trained models, each tailored to its respective dataset. Subsequently, these models were combined to enhance robustness and accuracy.

### Renal mass segmentation

A 3D U-Net network architecture was used to develop the automated segmentation model [9]. The segmentation model was designed to segment not only the renal tumors but also the kidney itself, ensuring that the renal masses could be accurately identified within the broader anatomical context. The kidney segmentation was conducted in an automated manner using the same 3D U-Net-based model, without any manual intervention. The network was trained using stochastic gradient descent with a batch size of 8. A combination of cross-entropy loss and Dice loss was employed as the training loss function, utilizing the Adam optimizer with an initial learning rate of 5e − 4. Each layer consisted of a 3D convolution with 3 × 3 × 3 kernels, followed by leaky ReLU activations [10] and instance normalization. Model training was performed on four GeForce RTX 3090 GPUs using the TensorFlow framework.

### Renal tumor classification features extraction

From the image datasets (precontrast, arterial, and delayed phases) for each patient, we extracted 342 imaging features, which were composed of both radiomic and conventional radiological features. Among these features, 321 radiomic features suggested by Joost et al. [11] were extracted via an open-source platform the pyradiomics library. This library includes features such as the size and shape of the region of interest (ROI), volume and compactness, sphericity of the tumor, distribution of voxel intensities within the tumor, and textural features. For the remaining features, 21 conventional radiology features based on differences in the biology and imaging characteristics of the tumor subtypes [12, 13] were extracted, consisting of HU intensity dynamics features, such as the mean intensity and minimum/maximum intensity, as well as calculated features such as standard deviations and difference ratios of intensity.

### Renal tumor classification

After tumor segmentation, radiomic and conventional radiology features were extracted from the segmented areas using deep learning models. These features were then used to train classifiers to distinguish renal tumor subtypes, utilizing logistic regression (LR), support vector classification (SVC), random forest classifier (RF) [14], and decision tree classifier (DT). To refine the dataset, significant features were selected from the extracted set using a recursive feature elimination with cross-validation (RFECV) algorithm [15]. Finally, the selected features were used as inputs for random forest models to build the classification models. Random Forest (RF) was particularly well-suited for this study due to its ability to handle high-dimensional data, mitigate the risks of overfitting, and provide feature importance measures, which made it ideal for capturing complex patterns and accurately classifying renal tumor subtypes.

## Results

### Performance of the automated segmentation model

**Fig. 2:**
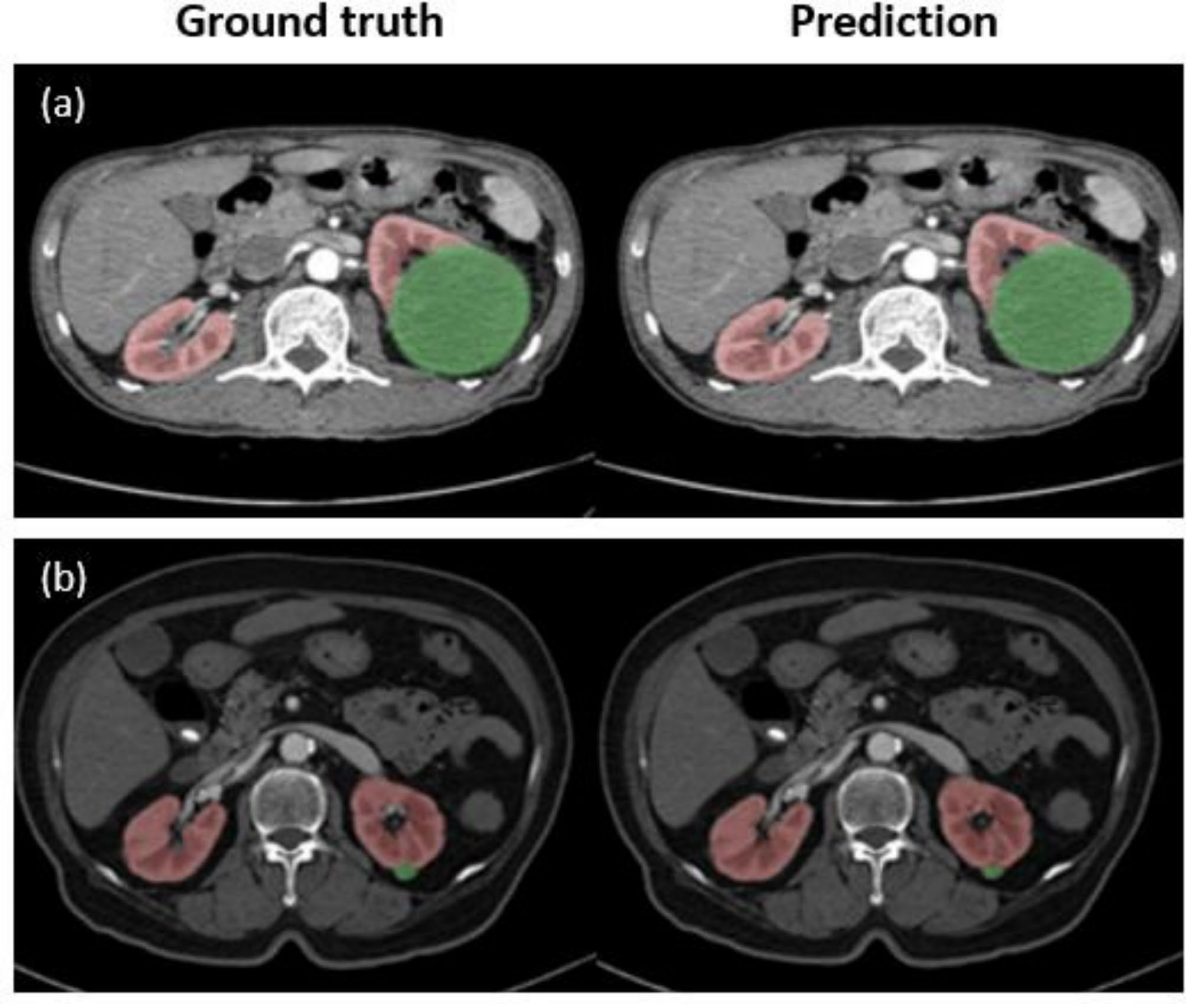
Performance of the automated tumor segmentation model for (**a**) kidneys and renal cell carcinoma (RCC) > 4 cm in size and (**b**) ≤ 4 cm in size

The performance of the automated segmentation model was evaluated by classifying tumors into large (≥ 4 cm) and small (< 4 cm) groups. The model performance results for the kidney and tumor regions are shown in Fig. 2. For large masses (≥ 4 cm), the Dice similarity coefficient, which defines the performance of the model, was 0.92 (95% confidence interval [CI], 0.82–0.95) for the kidney and 0.83 (95% CI, 0.76–0.89) for the tumor regions. By comparison, the performance of the model was low for masses < 4 cm, with Dice similarity coefficients of 0.84 (95% CI, 0.76–0.88) for the kidney and 0.65 (95% CI, 0.53–0.71) for the tumor regions.

### Performance of the classification model

The performance of the classification model was evaluated via conventional radiological and radiomic features, the prediction mask area, and a combination of these features to determine whether model performance could be further improved.

#### Performance of the classification model on the basis of tumor size

**Fig. 3:**
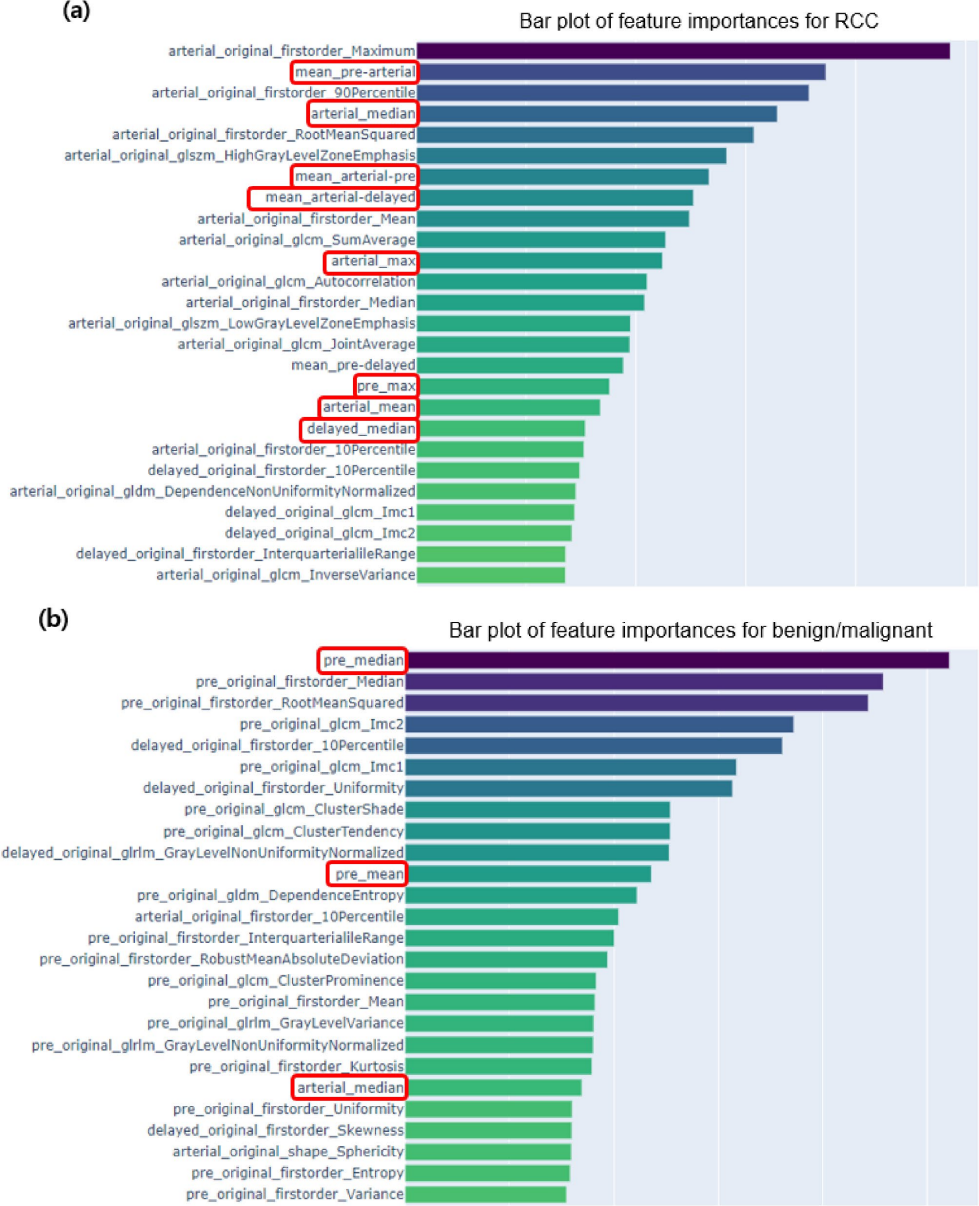
Features of importance for (**a**) classification of renal cell carcinoma (RCC) subtypes and (**b**) differentiation of benign and malignant tumors. The features in red boxes are derived from Hounsfield Units (HUs), such as the mean intensity, minimum, and maximum values and difference ratios, as well as changes in intensities between phases

The performance of the classification model by tumor mass size is presented in Table 2. For tumors larger than 4 cm, the accuracy of combining radiomics and conventional radiological features was 0.77 (95% CI, 0.69–0.86), which was higher than that of 0.74 (95% CI, 0.63–0.82) with radiomics and 0.67 (95% CI, 0.55–0.75) with conventional radiological features alone. In comparison, for tumors < 4 cm, the combination of radiomic and conventional radiology features did not yield an improvement in classification accuracy. Overall, considering tumors of all sizes, the classification accuracy was greater when the combination of radiomic and conventional radiology features was used, with an ACC of 0.72 (95% CI, 0.64–0.82), than when radiomic features of 0.70 (95% CI, 0.64–0.77) and conventional features of 0.63 (95% CI, 0.54–0.80) were used alone. Additionally, Fig. 3 shows that conventional radiological features, such as changes between the mean HU and different phases, have a significant impact on the subtype classification of renal tumors.

**Table 2:** Comparison of the performance of the automated segmentation model for the different image features used for tumors of different sizes and all renal cell carcinoma (RCC) subtypes

Feature Method	Size	ACC (95% CI)	PPV (95% CI)	NPV (95% CI)
Radiomics	≤ 4 cm	0.68 (0.58–0.78)	0.69 (0.60–0.81)	0.62 (0.45–0.78)
Conventional radiology		0.60 (0.50–0.71)	0.61 (0.48–0.73)	0.53 (0.33–0.73)
Radiomics + Conventional		0.68 (0.59–0.79)	0.70 (0.56–0.81)	0.64 (0.46–0.80)
Radiomics	> 4 cm	0.74 (0.63–0.82)	0.73 (0.60–0.84)	0.76 (0.57–0.90)
Conventional radiology		0.67 (0.55–0.75)	0.66 (0.53–0.78)	0.61 (0.42–0.78)
Radiomics + Conventional		0.77 (0.69–0.86)	0.79 (0.65–0.89)	0.73 (0.56–0.86)
Radiomics	All RCCs	0.70 (0.64–0.77)	0.70 (0.64–0.76)	0.85 (0.76–0.89)
Conventional radiology		0.63 (0.54–0.80)	0.64 (0.44–0.81)	0.81 (0.68–0.84)
Radiomics + Conventional		0.72 (0.64–0.82)	0.74 (0.61–0.85)	0.69 (0.51–0.83)

ACC, average coverage criterion; PPV, positive predictive value; NPV, negative predictive value; CI, confidence interval

#### Performance of the benign/malignant classification model for small renal masses

As shown in Table 3, the classification of benign and malignant SRMs via radiomic features yielded an ACC of 0.83 (95% CI, 0.74–0.90), with little improvement in accuracy when the combination of radiomic and conventional radiological features was used, with an ACC of 0.85 (95% CI, 0.74–0.90). Identification of the features important for the classification (Fig. 3b) indicated that conventional radiology features, such as the median HU of the pre- or arterial phase, had an impact on the differentiation of benign and malignant SRMs.

**Table 3:** Comparison of the performance of the classification model for the different image features used to differentiate benign and malignant tumors

Feature Method		ACC (95% CI)	PPV (95% CI)	NPV (95% CI)
Radiomics		0.83 (0.74– 0.90)	0.83 (0.70– 0.92)	0.81 (0.64– 0.92)
Conventional radiology		0.73 (0.64– 0.82)	0.74 (0.61– 0.85)	0.69 (0.51– 0.83)
Radiomics + Conventional		0.85 (0.74– 0.90)	0.84 (0.71– 0.92)	0.85 (0.69–0.95)

ACC, average coverage criterion; PPV, positive predictive value; NPV, negative predictive value; CI, confidence interval

## Discussion

In this study, we developed an end-to-end pipeline for the automated segmentation and classification of renal tumor subtypes in clinical practice on the basis of deep learning and ML using both radiomics and conventional radiological features either alone or in combination. Using voxel-level segmented CT volumes along a continuum of imaging phases—precontrast, arterial, and delayed phases—the performance of both the automated segmentation and the classification models was effective. Our model addresses an important clinical issue, namely, an increase in the incident detection of renal masses owing to the increased use of novel, noninvasive imaging methods for kidney disease [16], such as 99mTc-sestamibi single-photon emission or radiolabeled girentuximab CT, and multiparametric magnetic resonance (MR) imaging. Our model uses CT, which is consistent with clinical practice, where multiphase abdominal CT is accepted as the gold standard imaging modality for the detection and differentiation of renal tumors owing to its superiority over ultrasound and wider accessibility than MR imaging (MRI) [17].

Although the ML model of radiomic features alone showed some degree of diagnostic efficacy, several studies have combined radiomic features with other types of parameters to improve outcomes and integration into clinical practice. Nassiri et al. [18] further evaluated the use of clinical factors such as age, sex, smoking history, obesity, symptoms, and comorbidities; incorporating these patient demographic data into a radiomics-based predictive model did not improve the diagnostic yield. Yin et al. [19] also utilized multiomics datasets, such as clinical tumor-related parameters, including TNM stage; Fuhrman grade; and tumor size, mRNA, and microvascular density, derived from positron emission tomography (PET) and MR.

**Fig. 4:**
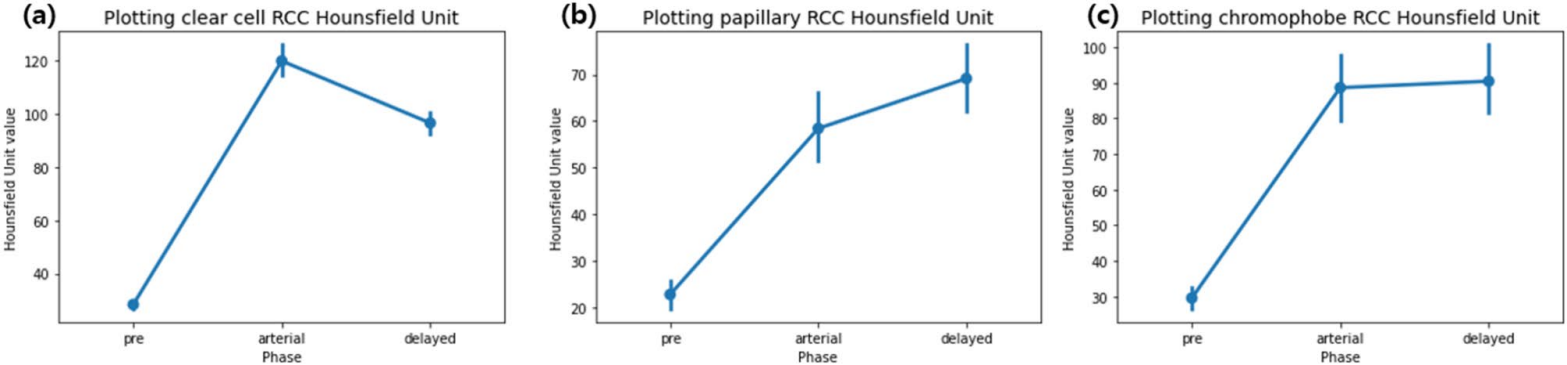
Variation in Hounsfield Units (HUs) values for different types of tumors. Shown are the change in intensity between the precontrast and the delayed phases for: (**a**) clear cell renal cell carcinoma (RCC), (**b**) papillary RCC, and (**c**) chromophobe RCC

A novel aspect of our ML model is the inclusion of both contrast-enhanced and dynamic pattern-related parameters in combination with radiomic features, which improved the efficacy of the model and, therefore, its potential usefulness in practice. Contrast-enhanced features offer intuitive tumor images, as well as descriptions of the arterial blood supply and flow into renal tumors [12, 13]. Contrast enhancement patterns familiar to clinicians, including uroradiologists, which were included in our model, are shown in Fig. 4. There is evidence that a large change in intensity (HU value) between the arterial and delayed phases is usually indicative of clear cell RCC, whereas a small change is indicative of a nonclear cell type. Therefore, the diagnosis of clear-cell RCC is characterized by high HU values in the arterial phase and decreased HU values in the delayed phase. On the other hand, for papillary RCCs, contrast enhancement is generally poor and gradually increases from the precontrast phase to the delayed phase. Chromophobe RCCs show an intermediate degree of change between clear-cell RCCs and papillary RCCs. Using these HU intensity values, we created 21 different parameters by extracting the mean, standard deviation, minimum, and maximum values for each phase, as well as including the between-phase change in intensity. Shehata et al. [20] used two contrast-enhanced pattern features, namely, wash-in and wash-out slopes, in a small number of patients [16]. Roussel et al. suggested the use of a similar parameter, namely, the arterial-to-delayed intensity ratio. However, according to our analysis of the 21 HU intensity, dynamic-related features, we identified the mean or median intensity of the delayed phase as being a more powerful feature to consider than either the wash-in or wash-out slope features were (Fig. 4). This finding is of clinical relevance, highlighting the importance of using HU intensity-related features for interpreting the nature of renal masses and yielding a more powerful ML model.

On the basis of the above evidence, the contrast-enhanced features in the arterial phase, which provided the clearest delineation of tumors, were used as the reference template for coregistration in the precontrast and delayed phases to improve the performance of the classification model for differentiating RCC subtypes. This improved the clinical usefulness of our model. Unlike previous studies, our automated segmentation process made it possible to automatically register segmentation information in other phases, reducing the burden of labeling in each phase and reducing the computational cost of the model.

The limitations of our study need to be acknowledged. First, the samples used in this study may not fully reflect clinical practice, as we included only three types of RCC and one benign tumor (AML). We do note that these are the most commonly observed types of RCCs (> 99%) worldwide [21] and the most common benign tumors in Eastern countries [22, 23]. Moreover, the study population was enrolled from the medical center prospective data cohort, consecutively, as previously described [7], which decreased generalizability but may have minimized study bias. Accordingly, we believe that our study provides practical insights and assistance for real-world scenarios for the classification of RCCs. Second, although some cases of fat-poor AML were included, we did not focus specifically on the differentiation of this subtype of AML, which is a diagnostically challenging situation owing to the limited number of cases. As noted by Firouzabadi et al. [24] in their systematic review, previous CT radiomics studies aimed at distinguishing fat-poor AML from clear cell RCC also consistently lacked sufficient numbers of fat-poor AML cases, including only 10 to 50 patients as in our study. To address this limitation, we have established the complete ML pipeline from automated segmentation of the renal mass to the final diagnosis, to easily perform a study with a substantially larger sample size by utilizing multicenter databases. Third, in the supplementary observations (Supplementary 1), distinguishing between cysts and renal tumors was difficult. Additionally, for a tumor size < 1 cm, the average Dice coefficient decreased to 0.43, indicating lower segmentation accuracy. Fourth, the tube voltage used in our institution varied based on the patient’s body mass index (BMI): 80 kVp was used for patients with a normal BMI, while 120 kVp was applied for those with a high BMI (> 25 kg/m²). This adjustment aimed to balance radiation dose and image quality. However, this variation in tube voltage represents a limitation of our study, as it may have influenced the values of radiomic features, as suggested by previous research [25]. Finally, external validation of our results is needed in a large patient population. To address this limitation, we are planning a multicenter study to validate our findings.

## Conclusions

We developed an effective and practical end-to-end framework for the classification of renal tumor subtypes on the basis of machine learning methods, which demonstrated promising results in renal tumor segmentation and classification. The model effectively distinguished between tumor and nontumor regions while accurately classifying renal tumor subtypes, offering deeper insight into tumor characteristics.

## Electronic supplementary material

Below is the link to the electronic supplementary material.

Supplementary Material 1

## Data Availability

The data sets generated during and/or analyzed during the current study are not publicly available due to retrospectively collected data but are available from the corresponding author on reasonable request.

## Abbreviations

CT: Computed tomography
RCC: Renal cell carcinoma
HU: Hounsfield units
AML: Angiomyolipoma
ML: Machine learning
LR: Logistic regression
SVC: Support vector classification
RF: Random forest classifier
RFECV: Recursive feature elimination with cross-validation
MR: Magnetic resonance
PET: Positron emission tomography
