# Supplementary material for: Fully automated segmentation and classification of renal tumors on CT scans via machine learning
Source: BMC Cancer. 2025 Jan 29;25:173. doi: 10.1186/s12885-025-13582-6 (PMC11781067; doi:10.1186/s12885-025-13582-6)
Supplement: Supplementary file 1 — Supplementary Material 1 [file 12885_2025_13582_MOESM1_ESM.docx]

**Supplementary**

**Supplementary 1.** Results for a failed case of automatic segmentation and tumor classification. Shown are (a) the difficulties of the classification in distinguishing between cysts and renal tumors (yellow arrows) and (b) of the lower accuracy of the automatic segmentation model for tumors <1 cm in size.


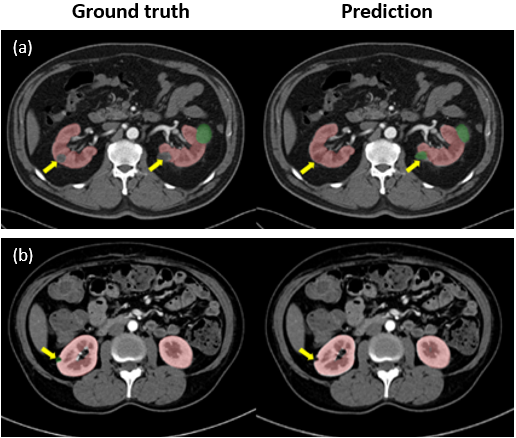


**List of conventional radiological features**

| **Feature** | **Description** |
| --- | --- |
| Hounsefield Unit mean | Mean HU Values for Arterial, Pre-contrast, and Venous Phases(CT 3 Phases) |
| Hounsefield Unit std | Standard Deviation of HU Values for CT 3 Phases |
| Hounsefield Unit minimum | Minimum HU Values for CT 3 Phases |
| Hounsefield Unit maximum | Maximum HU Values for CT 3 Phases |
| Hounsefield Unit median | Median HU Values for CT 3 Phases |
| Hounsefield Unit subtraction | Differences in HU Values Across the CT 3 Phases |
| Artray - Pre contrast / Artray | Relative Proportion of Arterial Phases to Pre-contrast Phases |
| Artray - Pre contrast / Pre contrast | Relative Proportion of Pre-contrast Phases to Arterial Phases |
| Venous - Artray / Venous | Relative Proportion of Arterial Phase to Venous Phase |
| Venous - Artray / Artray | Relative Proportion of Venous Phase to Arterial Phase |
| Venous - Pre contrast / Venous | Relative Proportion of Pre-contrast Phase to Venous Phase |
| Venous - Pre contrast / Pre contrast | Relative Proportion of Venous Phase to Pre-contrast Phase |
